# A Multisource Process Evaluation of a Community-Based Healthy Lifestyle Programme for Child and Adolescent Obesity †

**DOI:** 10.3390/children11020247

**Published:** 2024-02-15

**Authors:** Yvonne C. Anderson, Cervantée E. K. Wild, Catherine A. Gilchrist, Paul L. Hofman, Tami L. Cave, Tania Domett, Wayne S. Cutfield, José G. B. Derraik, Cameron C. Grant

**Affiliations:** 1 Department of Paediatrics: Child and Youth Health, Grafton Campus, University of Auckland, Private Bag 92019, Auckland 1142, New Zealand; cervantee.wild@auckland.ac.nz (C.E.K.W.); j.derraik@auckland.ac.nz (J.G.B.D.);; 2 Curtin Medical School, Faculty of Health Sciences, Curtin University, Bentley, WA 6102, Australia; 3 Telethon Kids Institute, Perth Children’s Hospital, Nedlands, WA 6009, Australia; 4 Child and Adolescent Community Health, Child and Adolescent Health Service, Perth, WA 6009, Australia; 5 Liggins Institute, University of Auckland, Auckland 1142, New Zealand; p.hofman@auckland.ac.nz (P.L.H.);; 6 Starship Children’s Hospital, Auckland District Health Board, 2 Park Road, Grafton, Auckland 1023, New Zealand; 7 Cogo Consulting, 58 Surrey Crescent, Grey Lynn, Auckland 1141, New Zealand; tania@cogo.co.nz

**Keywords:** access, evaluation, health care quality, multidisciplinary intervention, overweight, paediatric, programme, referral, service, stakeholder

## Abstract

Whānau Pakari is a healthy lifestyle assessment and intervention programme for children and adolescents with obesity in Taranaki (Aotearoa/New Zealand), which, in this region, replaced the nationally funded Green Prescription Active Families (GRxAF) programme. We compared national referral rates from the GRxAF programme (age 5–15 years) and the B4 School Check (B4SC, a national preschool health and development assessment) with referral rates in Taranaki from Whānau Pakari. We retrospectively analysed 5 years of clinical data (2010–2015), comparing referral rates before, during, and after the Whānau Pakari clinical trial, which was embedded within the programme. We also surveyed programme referrers and stakeholders about their experiences of Whānau Pakari, analysing their responses using a multiple-methods framework. After the Whānau Pakari trial commenced, Taranaki GRxAF referral rates increased markedly (2.3 pretrial to 7.2 per 1000 person-years), while NZ rates were largely unchanged (1.8–1.9 per 1000 person-years) (*p* < 0.0001 for differences during the trial). Post-trial, Taranaki GRxAF referral rates remained higher irrespective of ethnicity, being 1.8 to 3.2 times the national rates (*p* < 0.001). Taranaki B4SC referrals for obesity were nearly complete at 99% in the last trial year and 100% post-trial, compared with national rates threefold lower (31% and 32%, respectively; *p* < 0.0001), with Taranaki referral rates for extreme obesity sustained at 80% and exceeding national rates for both periods (58% and 62%, respectively; *p* < 0.01). Notably, a referral was 50% more likely for referrers who attended a Whānau Pakari training half-day (RR = 1.51; *p* = 0.009). Stakeholders credited the success of Whānau Pakari to its multidisciplinary team, family-centred approach, and home-based assessments. However, they highlighted challenges such as navigating multidisciplinary collaboration, engaging with families with complex needs, and shifting conventional healthcare practices. Given its favourable referral trends and stakeholder endorsement, Whānau Pakari appears to be a viable contemporary model for an accessible and culturally appropriate intervention on a national and potentially international scale.

## 1. Introduction

Avoidable health inequities primarily stem from how healthcare systems are structured, affecting access to services [1]. This uneven distribution of healthcare services is a crucial societal determinant of health [1]. All sectors must collaborate to effectively address these societal determinants, addressing funding ‘silos’ to enact change [1]. The *World Health Organization (WHO) Commission on Social Determinants of Health* advocates that the highest levels of government take responsibility for health inequities [1]. This approach ensures that health considerations, including those related to childhood obesity, are integrated across all policy areas [2].

Health inequities are associated with childhood obesity, globally and in Aotearoa/New Zealand (NZ). Approximately 13% of NZ children aged 2–14 years are affected by obesity, with those who identify as Māori (NZ Indigenous) or Pacific peoples, and those from the most socioeconomically deprived areas most affected [3]. These observations corroborate international data showing prevalent health disparities between Indigenous and non-Indigenous populations [4].

There is a fragmented delivery of multidisciplinary intervention programmes to achieve healthy lifestyle changes in childhood and adolescence in NZ, despite these programmes remaining internationally recommended best practice [2,5,6]. The creation of any multidisciplinary interventions to address childhood obesity should consider the needs of those most overrepresented in obesity statistics [2].

In this context, the Green Prescription Active Families (GRxAF) programme has been one of the most common interventions in NZ, addressing childhood obesity and physical inactivity [7]. Regional Sports Trusts deliver sports and active recreation programmes in their communities and run the GRxAF programme in some centres nationally [7]. An audit of the GRxAF programme in the Taranaki region of NZ showed that participation was associated with lifestyle changes in some children and their families [7]. However, such changes were not universal, and the needs of some families were not met. Programme delivery for Māori *whānau* (wider family unit) and accessibility of the service regionwide were areas identified for improvement [7]. On this basis, programme development focused on enhancing engagement and reach for those children most affected by obesity, thereby addressing inequity [7].

Whānau Pakari is a home-based, family-centred assessment and intervention healthy lifestyle programme that evolved from the need for improved access and appropriateness of healthy lifestyle services in the Taranaki region of NZ [8]. The Whānau Pakari programme replaced GRxAF based on the audit findings of issues with access and the need to improve equity outcomes [7]. A randomised clinical trial (RCT) embedded within the community programme assessed Whānau Pakari’s effectiveness. Twelve-month outcomes found Māori participation at 47% (c.f. population prevalence 28%) [9] and a 28% representation from those living in the most deprived quintile of households in Taranaki (c.f. population prevalence 15%) [10]. At the 12-month follow-up, participants displayed a similar change in body mass index standard deviation score (BMI SDS) from baseline in both the low-intensity control group (home-based assessment with advice; −0.12 [95% CI −0.20, −0.03]) and the high-intensity intervention group (same home-based assessment with weekly group activity sessions; −0.10 [−0.19, −0.02]) [10]. However, participants in the intervention group who attended ≥70% of the weekly sessions experienced a BMI SDS reduction markedly greater than those who attended <70% (−0.22 [−0.36, −0.09] versus −0.04 [−0.14, 0.05] respectively; *p* = 0.04) [10]. This reduction persisted at the two-year follow-up (≥70%: −0.22 [−0.38, −0.06] versus <70%: 0.09 [−0.02, 0.20]; *p* = 0.002) [11], but BMI SDS returned to baseline levels at the five-year follow-up [12]. Nonetheless, at the five-year follow-up, there were persistent improvements in health-related quality of life and increased water intake in both groups, and a reduced sweet drink intake in the high-intensity intervention group [12].

Importantly, an economic evaluation showed that the Whānau Pakari programme was cost effective at 12 months compared with the previous conventional model of care [13]. After recruitment for the RCT ended in August 2014, an interim service model commenced, which became the agreed service model for a multidisciplinary healthy lifestyle programme for the Taranaki District Health Board (DHB) in December 2016 [14]. The programme continues to receive referrals in a ‘business-as-usual’ service delivery model [14]. 

We have described the Whānau Pakari programme elsewhere [8]. In brief, the programme includes not only the intervention programme of weekly sessions, but also a home-based assessment to ensure weight-related comorbidities are identified and addressed in all participants [8]. This provides a ‘one-stop-shop’ model, ‘demedicalising’ obesity, where hospital visits are not required, and is a family-focused intervention. The 12-month programme within the trial included baseline, 6-month, and 12-month home-based assessments for all participants with the healthy lifestyle coordinator (a health professional trained in weight-related assessments), one home visit by the dietitian and/or physical activity coordinator, and weekly intervention sessions during the school term for 12 months [8]. The same programme is provided currently, although it now consists of 6 months of weekly sessions during the school term. The clinical assessments included medical history and lifestyle (i.e., eating behaviour and physical activity) screening, psychological screening, and physical examination. Paediatrician oversight for the medical assessments was provided at multidisciplinary team meetings, as well as psychological, dietetic, and physical activity coordinator oversight and support [8]. Weekly intervention sessions comprised various components: dietitian-led activities, including virtual supermarket tours, cooking demonstrations, guidance on growing vegetables, portion sizes, and label reading; psychology sessions focused on self-esteem, parenting strategies, family dynamics, and sleep; and physical activity sessions featured a range of games and sports [8].

While research often concentrates on weight outcomes to assess the efficacy of obesity interventions, conducting multisource process evaluations alongside these efficacy studies is equally important. Such evaluations help determine how these interventions impact the broader societal context in which they operate [15]. The primary goal of this multisource evaluation was to gauge the impact of the Whānau Pakari programme within the community, including changes in referral patterns over time. Second, we sought to compare the referrals to Whānau Pakari with those made to a similar national programme (GRxAF) and referrals for obesity from the B4 School Check (B4SC—a free health and development screening for preschoolers) [16]. Third, we aimed to determine referrer and stakeholder experience of and satisfaction with the programme, given this was a novel, multidisciplinary obesity assessment and intervention programme for NZ. Participant and parent/caregiver experience from focus groups has been reported elsewhere [17].

## 2. Materials and Methods

We used a multiple-methods framework to examine Whānau Pakari’s effectiveness in the community [14]. The Whānau Pakari programme supports children and young people 4–16 years of age, encompassing the age ranges of Taranaki’s B4SC and the GRxAF nationally [8]. Taranaki is a mixed urban-rural region of NZ, with 23,127 children aged 0–15 years in the 2013 census [9]. Participants in the Whānau Pakari RCT were recruited in 2012–2014 [8]. For the present study, we audited national data covering three periods [14]:

- July 2010–December 2011: 18 months before the start of Whānau Pakari
- January 2012–August 2014: 31 months during the trial
- September 2014–September 2015: 13 months after trial completion

### 2.1. National Comparisons

#### 2.1.1. Green Prescription Active Families (GRxAF)

The GRxAF programme, supporting children and adolescents aged 5–18 years, operated in Taranaki from 2007 to 2011 [7]. It aimed to improve physical activity and nutrition in the community [7]. Although it was not originally intended as an intervention for childhood obesity per se, most referrals were related to weight concerns [7]. The GRxAF programme has a family-centred approach, including home visits and weekly activity sessions lasting up to 12 months [7].

To compare with the national GRxAF data (covering ages 5–15 years), we included only participants within the same age range from the Whānau Pakari cohort [14]. The GRxAF programme was provided by 15 of NZ’s 20 DHBs from January 2010 until December 2015 (with many regions continuing to the present day), while the Taranaki DHB offered Whānau Pakari from January 2012. Using maps of the DHBs and geographical regions, we defined the regional GRxAF boundaries [14]. The eligible paediatric population for GRxAF referrals was identified based on 2013 NZ Census data, limited to young people aged 5–15 years in DHB regions offering GRxAF [18]. This population included those in the 5–9- and 10–14-year age groups, as well as 20% of the 15–19-year age band [19]. Auckland, Canterbury, South Canterbury, Waitematā, and West Coast DHBs were excluded, as they did not offer GRxAF [14].

Referral rates were calculated using GRxAF quarterly report data to the Ministry of Health [14], covering total, Māori, and NZ European referrals across NZ and specifically for Taranaki (January 2012 onwards for Whānau Pakari), using the following formula:

$$GRxAF\;referral\;rate\;=\;\frac{n}{\left(\frac{m}{12}\right)}\times \frac{1}{N}\times 1000$$

where *n* was the number of GRxAF referrals made over a given number of months (*m*), and *N* was the background population, with the GRxAF referral rate expressed as *n* per 1000 person-years.

Note that we could not ascertain the eligible paediatric population affected by obesity because, during this period, some GRxAF programmes accepted children with a healthy weight but who were physically inactive [14]. Self-reported and self-prioritised ethnicities were determined for all GRxAF participants, but data before 2012 did not include ethnicity data for all ‘referrals’. Hence, ethnicity data from January 2010 to December 2011 were only used for those ‘engaged’ with the programme. Finally, we calculated the total eligible Māori and NZ European populations within the defined paediatric population and the corresponding groups in Taranaki [18].

#### 2.1.2. B4 School Check (B4SC)

The B4SC is a free national health and development screening for children in their fourth year [18]. From July 2010 to June 2015 (review period), it was expected that all children with a BMI exceeding the 99.6th percentile (i.e., with ‘extreme’ obesity) would be referred for further assessment and potentially ongoing weight monitoring [14,20]. In Taranaki, starting from January 2012, those referrals were directed to Whānau Pakari [10].

The B4SC data for the review period were separated into year bands. We recorded the number of children identified within the overweight (>91st to ≤98th percentiles), obesity (>98th to ≤99.6th percentiles), and ‘extreme’ obesity (>99.6th percentile) categories for Taranaki and nationally, as per the NZ Ministry of Health "weight-height BMI” conversion charts [20,21,22]. For each outcome, the total number referred was determined; from July 2012, this included ‘referred’ and ‘advice given’, a new category added by the Ministry of Health and included in the total referred [14]. Rates for obesity and extreme obesity were calculated as follows:

$$B4SC\;referral\;rate\;\left(\%\right)\;=\;\frac{n}{\left(\frac{m}{12}\right)}\times \frac{1}{N}\times 100$$

where *n* was the number of B4SC referrals for the BMI group made over a given number of months (*m*), and *N* was the number of children assessed in the B4SC in the same period with the corresponding BMI status.

### 2.2. Whānau Pakari Evaluation

#### 2.2.1. Referrer Satisfaction

We created a referrer survey using SurveyMonkey (SurveyMonkey Inc., San Mateo, CA, USA), which was beta-tested by five potential referrers before distribution. On 19 November 2015, we distributed a questionnaire to 317 identified potential referrers within the region, setting the response deadline for 18 December 2015. These potential referrers encompassed a diverse group of healthcare professionals and specialists: general practitioners (primary healthcare physicians), public health nurses, paediatricians, paediatric registrars, house officers, dietitians, *kaiāwhina* (Māori healthcare workers), orthopaedic surgeons, general surgeons, otorhinolaryngologists, school counsellors, psychologists, and adult physical activity coordinators (who were seeing the parents of eligible children) [14]. Referrers received weekly reminder emails.

#### 2.2.2. Stakeholder Satisfaction

Cogo Consulting, a research consultancy firm in Auckland (NZ), was hired as an external evaluator to conduct a focused process evaluation of Whānau Pakari. Its evaluation centred on assessing the acceptability of the service delivery model to various project partners and stakeholders [14]. These stakeholders included programme delivery team members and leads, governance/advisory group members, funders and planners, subject matter experts, ministry officials, referrers, and research associates [14]. An online survey was developed using the web-based SurveyGizmo (Boulder, CO, USA) to gather stakeholder feedback [14]. The survey invited project stakeholders to share their experiences with the Whānau Pakari programme (but not the RCT), offering insights into what aspects worked and identifying potential barriers to success [14]. Cogo Consulting invited 24 stakeholders to complete the online survey anonymously. The survey used five-point Likert scales to collect quantitative data and open-text comment fields to collect qualitative data.

### 2.3. Data Analyses

For each period of interest (before, during, and after the Whānau Pakari RCT), GRxAF and B4SC referral rates were compared between Taranaki and the rest of NZ using Fisher’s exact tests in Minitab v21.4 (Pennsylvania State University, State College, PA, USA). In addition, we examined the association between attendance at a Whānau Pakari trainee half-day and the likelihood of making a referral using the *risk ratio* function from the *epitools* package in R v4.3.2 [23], which is reported as the relative risk (RR) and its 95% confidence interval (CI). All statistical tests were two-sided at a 5% significance level.

Qualitative analysis was performed on referrer and stakeholder feedback from the online survey. Open-text responses underwent descriptive qualitative analysis using inductive and semantic coding by Cogo Consulting [24].

### 2.4. Ethics

Under section 3 of the *Standard Operating Procedures for Health and Disability Ethics Committees v.3.0*, clinical audit studies in New Zealand are not required to be reviewed by the Committees [25]. Nonetheless, this study complied with all the principles set by the *National Ethical Standards for Health and Disability Research and Quality Improvement 2019* [26], as required for all health researchers under the NZ *Code of Health and Disability Services Consumers’ Rights 1996* [27]. However, in this study, we also report a subset of data from the Whānau Pakari RCT as part of our secondary analysis. The Whānau Pakari trial was registered with the *Australian New Zealand Clinical Trials Registry* (ANZCTR; registration number: 12611000862943) and received ethics approval from the *Central Health and Disability Ethics Committee* (Reference: CEN/11/09/054).

## 3. Results

### 3.1. National Comparisons

#### 3.1.1. GRxAF

Prior to the Whānau Pakari RCT, there were only marginal differences in GRxAF referral rates between Taranaki and the rest of NZ (Table 1). However, following Whānau Pakari’s inception, GRxAF referral rates were nearly 4-fold higher in Taranaki overall and for both Māori and NZ European children and young people (Table 1). While the magnitude of the difference decreased after trial recruitment ended, referral rates in Taranaki were still 1.8 to 3.2 times higher than in the rest of NZ (Table 1).

#### 3.1.2. B4 School Check

Prior to Whānau Pakari and during the transition period, referral rates for children identified as experiencing obesity or extreme obesity in the B4SC were similar in Taranaki and the rest of NZ (Table 2). However, during the Whānau Pakari RCT, referral rates in Taranaki were higher than in the rest of NZ, remaining so even after the RCT ended (Table 2). Notably, Taranaki B4SC referrals for obesity were nearly universal at 99% in the last trial year and 100% post-trial, compared with national rates 3-fold lower (31% and 32%, respectively; *p* < 0.0001), as all but one of the 162 children affected by obesity in Taranaki were referred from the B4SC (85/86 and 76/76, respectively) (Table 2). For extreme obesity, B4SC referral rates in Taranaki were sustained at 80% and exceeded national rates for both periods (58% and 62%, respectively; *p* < 0.01) (Table 2).

### 3.2. Evaluation of the Whānau Pakari Programme

#### 3.2.1. Referrer Survey

Out of 317 ‘potential’ referrers, we received 113 complete responses (36% response rate) from a wide range of professionals. Among the respondents, 106 (93%) were aware of the Whānau Pakari programme. The most common sources of their awareness were word of mouth (n = 30; 28%) and presentations by the Whānau Pakari team (n = 30; 28%). Additionally, 70 respondents (66%) had referred at least one individual to the Whānau Pakari programme. Reported reasons for non-referrals included: “I have not seen anyone eligible for referral to Whānau Pakari” (n = 12; 34%) and “I have not been looking for eligible patients/clients“ (n = 5; 14%).

Among survey respondents, 21 (20%) had participated in training half-day sessions conducted by the Whānau Pakari team. Forty respondents (37%) reported they were more frequently calculating BMI in their practice due to the Whānau Pakari programme. Further, 55 (52%) respondents indicated they were referring more children/adolescents identified as having overweight or obesity to the Whānau Pakari programme compared with other options available in Taranaki before the programme began in January 2012.

The Whānau Pakari programme increased awareness about childhood obesity as a health issue for 66 respondents (62%). In addition, 70 respondents (66%) were more likely to discuss obesity with their patients, clients, and/or families. Notably, respondents who attended one of the Whānau Pakari trainee half-day sessions were 50% more likely to refer children and adolescents to the Whānau Pakari programme than those who did not (RR = 1.51 [95% CI 1.21, 1.88]; *p* = 0.009) (Supplementary Table S1). Comments highlighting both the positives and the challenges experienced by referrers in this area of practice and their experiences of the Whānau Pakari programme are provided in Supplementary Table S2.

#### 3.2.2. Stakeholder Satisfaction

Of the total survey requests (n = 24), 18 stakeholders returned complete responses (75% response rate) with detailed feedback. The sample included a wide range of stakeholders, whose recommendations are presented in Supplementary Table S3.

Stakeholders considered the multidisciplinary composition of the delivery team critical to the Whānau Pakari programme’s success, as all 18 respondents stated the multidisciplinary composition of the team was ‘very’ or ‘somewhat important’. All roles within the multidisciplinary team were deemed ‘very important’ by most respondents (healthy lifestyles coordinator [100% agreement], physical activity coordinator [88%], dietitian [84%], psychologist [72%], paediatrician [67%]). Six respondents (33%) indicated a need for more professionals in the programme, specifically suggesting the inclusion of Māori health workers and general practitioners/primary care providers. The remaining 12 respondents (67%) reported the range of disciplines worked well.

Most respondents (n = 15; 83%) reported that the multidisciplinary team worked either ‘well’ or ‘very well’ together (the remaining three answered ‘don’t know’). Respondents identified key elements for success within the multidisciplinary team, which included collaboration, communication, different perspectives complementing each other, cohesive team structure, regular meetings, clearly defined responsibilities, excellent planning, and a positive working environment with mutual respect.

##### Home Visits

Home visits were a critical feature of the Whānau Pakari programme that aimed to reach and successfully engage with the target population groups. Sixteen respondents (89%) reported that home visits were ‘somewhat’ or ‘very important’ to the recruitment of participants and to inspire ongoing participation within the programme. The open-text responses emphasised this, with comments such as, “Without the home visits, calls, chasing of patients, we would not have a Whānau Pakari intake like we have”. Respondents also reported how important this was for seeing families ‘in their own reality’ and that it allowed the staff to assess the nearby food and physical activity environment, for example, “How far away from a park is their house, can they afford good quality food (…)”.

##### Importance of ‘Demedicalisation’ of the Programme

The ‘demedicalisation’ inherent to Whānau Pakari programme delivery (i.e., moving programme delivery from a clinical setting to home and community settings without compromising the quality of care) was considered ‘very’ or ‘somewhat important’ (n = 16; 89%) for recruiting participants from target population groups, and ‘very’ or ‘somewhat important’ (n = 15; 84%) for keeping participants engaged in the programme. The open-text comments highlighted that this approach was key to participant comfort, with one respondent stating that the ‘demedicalisation’ “removes a barrier for some families who would feel less comfortable navigating the hospital clinic visit of the conventional medical model”. Comments also suggested this model encouraged a sense of agency central to families’ engagement in the process. For example, one respondent highlighted the sense of ownership that the ‘demedicalised’ model facilitated: “If you move this *[hospital appointment]* away from hospital base—then you allow families to feel that sense of ownership. Otherwise, they will feel they ‘have to do as they are told’”.

##### Referral Process

The Whānau Pakari programme receives referrals from any health professional or school staff member, as well as self-referrals, to ensure there are no referral barriers. This referral process appeared to be working, as 66% of respondents (n = 12) stated the referral process worked ‘well’ or ‘very well’.

##### Cultural Responsiveness

A key Whānau Pakari objective was to offer a culturally appropriate and accessible programme for Māori. Regarding cultural responsiveness, stakeholders considered the programme design responsive to Māori, with 13 respondents (73%) describing it as either ‘extremely responsive’ or ‘very responsive’. Most respondents thought the programme was family-centred (‘extremely’ or ‘very’ n = 17, 95%). However, there was a reported need to improve Māori governance, with a wider spread of responses about programme responsiveness; ‘extremely’ or ‘very’ (n = 8; 45%), ‘neutral’ (n = 4; 22%), ‘somewhat’ or ‘not at all’ (n = 4; 22%), and ‘don’t know’ (n = 2; 11%). Recommended aspects for improvement were *marae*-based (traditional Māori meeting house) delivery, higher Māori representation in the delivery team, and a stronger Māori ‘lens’ concerning governance.

##### Balance between Clinical and Social/Community Needs

Most stakeholders reported that the programme effectively balanced the clinical, social, and community needs of participants alongside those of their families. Specifically, 13 respondents (73%) stated that the programme achieved this balance ‘well’ or ‘very well’, suggesting the current delivery and location were appropriately aligned to their needs.

##### Appropriateness of the Service Delivery Model

Most respondents thought the Whānau Pakari service delivery model was ‘highly appropriate’ or ‘appropriate’ (n = 16, 89%). When asked to reflect on how the Whānau Pakari programme had evolved since inception and what stakeholders would do differently, common elements arising from descriptive qualitative analysis were: more clarity around the importance of the clinical trial, a marae-based venue, Māori leadership of the programme, and a change in age group to enable age-appropriate content.

##### Success Factors, Barriers, and Transferability of the Whānau Pakari Programme

Most stakeholders reported in an open-ended question that the Whānau Pakari programme is a model that could be translated to other regions in NZ, with 11 (65%) giving an unqualified ‘yes’. A further 5 (30%) also agreed, but with the caveat that regional adaptation and increased Māori involvement are necessary.

##### Critical Success Factors and Challenges

When asked “What are the critical success factors in setting up a child obesity service?”, key elements identified in open responses were a multidisciplinary delivery team, family-friendly services that support engagement, and a home- or marae-based model. Other factors cited were effective marketing, strong community liaison, organisation buy-in/commitment, Māori engagement, multiple stakeholders involved in programme design and governance, clear programme objectives, and programme evaluation and feedback to referrers.

Respondents identified several challenges in establishing a childhood obesity programme. These included working in a multidisciplinary team, addressing complex-needs population groups, challenging traditional healthcare practices related to childhood obesity, and encountering difficulties engaging with some families or family members. Additional challenges reported were redefining perceptions of childhood obesity and its multiple determinants, securing broader support for a *Kaupapa Māori*-informed approach (respecting Māori philosophy, worldview, and cultural principles) within the existing healthcare model [28,29,30], and balancing clinical requirements with community needs. There was also a need for ongoing follow-up to assess the long-term population-level health impacts of the programme within the constraints of allocated funding. Prejudice against children affected by obesity, the cross-sectoral nature of the service not aligning with the funding models of individual organisations, and the necessity to tailor services for different age groups were also noted as important challenges.

## 4. Discussion

This evaluation of a multidisciplinary family-based intervention for children and adolescents with obesity showed that the programme was well received by stakeholders and referrers, with several identified benefits. Previous research has shown that Whānau Pakari achieved high participation rates, particularly from Māori and more socioeconomically deprived households [10]. The programme delivered higher overall referral rates for children with obesity (especially for Māori) compared with a similar national programme (GrxAF). Referral rates from the B4SC in Taranaki also exceeded the national figures during the study period, with the decline in post-trial referrals likely due to reduced promotion after the trial ended. Overall, referrer satisfaction was high, suggesting a positive impact of the Whānau Pakari programme in the community by raising awareness and facilitating conversations about childhood weight and healthy lifestyle changes. Stakeholders reported high satisfaction with the programme, providing actionable recommendations for ongoing programme development and identifying key elements for programme transferability.

Historically, the Whānau Pakari programme evolved from the GRxAF model, originally funded to serve children and adolescents aged 5–18 years [7]. Whānau Pakari was funded to accept referrals for ages 5–16 years, later expanding to 4–16 years to accommodate B4SC referrals, but the team recognised early on that the age range was too wide for effective delivery of activity sessions. Based on these insights and the RCT findings [10], the Whānau Pakari programme delivery was improved. It now offers sessions tailored for 6–12-year-olds and a workshop model for adolescents aged over 12 years, with pilot programmes for preschools also implemented [31,32].

Importantly, focus group discussions and in-depth interviews revealed that the marked increase in referral rates to the Whānau Pakari programme, as compared with prior programmes and clinics, can be attributed to its non-judgemental and non-stigmatising model with a ‘demedicalised’ approach [17,33]. To effectively engage with the populations most impacted by obesity, there is a clear need for programmes that are both accessible and community-based [10]. In this context, this study identified critical factors necessary to scale the Whānau Pakari model to other NZ regions and elsewhere:

- A multidisciplinary programme delivery team with access to the appropriate hospital records.
- A supportive, family-friendly, and culturally appropriate programme for all participants and their families.
- A home-based (or Indigenous health) model to maximise accessibility.
- A clinical lead in childhood obesity and healthy lifestyle changes for each region to provide clinical leadership for the multidisciplinary team.

In addition, we strongly recommend appointing a community champion in each region to engage with the community and provide leadership for the multidisciplinary team. Further, implementing a database or IT application to enhance communication and efficiency across multiple geographic locations is recommended. This would facilitate the seamless delivery of a programme that assesses weight-related comorbidities within a community-based healthy lifestyle programme, thereby enhancing the quality of care and eliminating the need for hospital visits [10,34]. Though not covered in our stakeholder survey, the absence of an IT platform would have substantially hindered the functionality of the Whānau Pakari programme in terms of multidisciplinary teamwork, participant assessments, team communication, and the ability to work remotely. Other factors identified as important included effective marketing, strong community liaison, organisation buy-in/commitment, Māori engagement, the inclusion of multiple stakeholders involved in programme design and at the governance level, clear programme objectives, ongoing programme evaluation, and regular feedback to referrers. A noted funding gap within the team has been support for Māori health workers, and it is recommended that this is addressed in future resource allocation for any multidisciplinary team working in NZ. These are all key considerations for the potential scaling of the Whānau Pakari framework.

A key strength of this study was the comprehensive feedback provided by stakeholders and referrers. This evaluation complemented the results from the Whānau Pakari RCT [10,11,12] by incorporating a multiple-methods framework, allowing for a more nuanced understanding of the findings. Additionally, it enabled comparisons with national data from the GRxAF and B4SC programmes during a period of policy changes. This comparative approach facilitated the evaluation of both pre- and post-policy changes, thus enabling an assessment of the potential impacts. Another significant strength of this study is its contribution to the limited literature on process evaluations of real-world clinical trials, particularly those assessing contemporary clinical models of care. While we also acknowledge the sample size limitations inherent to Taranaki’s relatively small population, this factor allowed for comprehensive analyses of obesity referrals in the region and, consequently, reliable assessments of Whānau Pakari’s impact on referral rates. Other limitations were the inconsistencies between the boundaries of DHBs and Regional Sports Trusts, which were occasionally conflicting. The survey response rate of referrers was also relatively low (as it encompassed all ‘potential’ referrers), which might have led to sample bias. Ethnicity data were prioritised at collection. However, the GRxAF programme only recorded ethnicity data for those engaged in the programme before 2012, resulting in incomplete data that should be interpreted with caution. Caution should also apply to the interpretation of national B4SC referral data, as many regions of NZ did not have an ‘obesity’ programme for referral during the study period. However, it was expected that all children identified as having extreme obesity (>99.6th percentile) would be referred to an intervention programme [20]. In addition, it was also presumed that all young people assessed with BMI ≥ 91st percentile would have been referred for obesity/weight issues, even though some might have been referred for other health reasons. Regrettably, the B4SC data did not contain this level of information. The B4SC introduced an ‘advice given’ referral group in 2011–2012, which we considered a referral. This category varied in usage, ranging from a referral to an in-house specialist team to the assessor giving nutritional advice during the assessment. Additionally, another B4SC category where the child was already under the care of a general practitioner and/or paediatrician for weight-related concerns (‘under care’) might have impacted referral rates, as we included these in our study. Thus, we might have overestimated the number of true referrals nationally for obesity. Lastly, Whānau Pakari welcomed but was unable to adequately assess the progress of Pacific peoples within the programme, who represent only 3% of the Taranaki population [9]. Pacific children are nearly four times more likely to experience obesity than their non-Māori and non-Pacific counterparts [3]. Thus, successful translation of such model to other NZ regions, particularly Auckland, would require community engagement and place-based consideration of the needs of Pacific children and families.

Based on our present findings and our previous research, Whānau Pakari remains a successful intervention programme for children and adolescents with obesity in Taranaki [10]. Implementing a similar model of care in other NZ regions and internationally could improve health outcomes for children and young people affected by obesity, aligning with many goals of the NZ Health Systems Reform [35]. While a successful approach in one region of NZ may not be immediately applicable in other areas or internationally, Whānau Pakari’s evidence-based framework provides a versatile structure that can be adapted and co-created with local communities elsewhere, utilising critical success factors from the Whānau Pakari model with a standardised digital application whilst honouring place-based and cultural considerations. Integrating well-designed digital technologies can facilitate the effective screening of weight-related comorbidities within such programmes, potentially lowering screening costs while ensuring a patient-centred approach [34]. For the ‘scaling-up’ of any intervention to be feasible and equitable, it must become fully integrated into the healthcare system with sufficient resources to ensure long-term, sustainable health benefits [36]. This level of integration has been successfully achieved regionally.

Ultimately, beyond short-term benefits, the evaluation and monitoring of obesity interventions are essential to ascertain their long-term effectiveness and relevance. For example, the *United States Centers for Disease Control and Prevention* (CDC) has developed *An Evaluation Framework for Obesity Prevention Policy Interventions* emphasizing a systematic approach to evaluating obesity interventions that accounts for the cyclical and complex nature of health policies [37]. This framework suggests that evaluations should consider not only the intended outcomes but also broader outcome measures alongside the potential for unintended adverse effects. This will ensure that public health interventions contribute positively without exacerbating existing disparities (such as health inequities) or creating new issues [37]. In the context of Whānau Pakari, as previously mentioned, the trial’s five-year follow-up revealed promising outcomes, namely sustained improvements in health-related quality of life, increased water consumption, and reduced sweet drink intake [12]. Although the initial reductions in BMI SDS were not maintained overall, there is evidence from large studies of an upwards BMI trajectory over time in young people with obesity [38,39,40]. Therefore, the absence of a mean BMI change overall at five years is notable, underscoring the importance of long-term follow-up of children and adolescents in the Whānau Pakari and other multidisciplinary programs.

## 5. Conclusions

The Whānau Pakari programme has garnered strong community buy-in, evidenced by both clinical quantitative measures after a 12-month intervention and qualitative outcomes within a multiple-methods evaluation framework. Stakeholders have recognised the programme’s framework as transferable, though they suggest place-based considerations such as regional modifications and appropriate engagement strategies tailored to specific population groups. Policymakers should explore the scalability of this model in the context of health system reforms. Integrating the Whānau Pakari approach with digital technology could serve as a foundational structure for the development of ‘by-community-for-community’ healthy lifestyle programmes. These could be potentially scaled across NZ and other countries.

## Figures and Tables

**Table 1:** Referral rates from the Green Prescription Active Families (GRxAF) programme in Taranaki and the rest of New Zealand, in relation to the Whānau Pakari randomised clinical trial and the ongoing programme.

Study Period	Date	Study Population	Taranaki	New Zealand	*p*-Value
N	NZ Census 2013	Overall	15,967	455,522	–
		Māori	4509	121,624	–
		NZ European	13,436	330,224	–
Before trial	Jul 2010–Dec 2011	Overall	56 (2.3)	1239 (1.8)	0.07
		Māori	12 (1.8)	310 (1.7)	0.88
		NZ European	24 (1.2)	243 (0.5)	<0.001
During trial	Jan 2012–Aug 2014	Overall	317 (7.2)	2427 (1.9)	<0.0001
		Māori	116 (9.4)	863 (2.6)	<0.0001
		NZ European	117 (3.2)	790 (0.9)	<0.0001
After trial	Sep 2014–Sep 2015	Overall	65 (4.1)	1050 (2.3)	<0.0001
(ongoing programme)		Māori	32 (7.1)	394 (3.2)	<0.001
		NZ European	28 (2.1)	326 (1.0)	<0.001

*N* is the size of the background population from the NZ Census 2013 data for a given study population, which was used as the denominator to calculate the referral rate. As the referral periods differed in length, for each study period and study population, the data provided are the number of referrals and the corresponding rate (in brackets) expressed as the number of referrals per 1000 person-years. *p*-values were derived from Fisher’s exact tests, with those statistically significant at *p* < 0.05 shown in bold.

**Table 2:** Referral rates from the B4 School Check for children with obesity (BMI 98–99.6th percentiles) and extreme obesity (>99.6th percentile) in Taranaki and the rest of New Zealand, in relation to the Whānau Pakari randomised clinical trial and the ongoing programme.

Period	Period	BMI Category	Taranaki	National	*p*-Value
Before the trial	Jul 2010–Jun 2011	Obesity	0/86 (nil)	21/2770 (1%)	>0.99
		Extreme obesity	2/70 (3%)	157/2253 (7%)	0.23
Transition period	Jul 2011–Jun 2012	Obesity	1/84 (1%)	74/2765 (3%)	0.73
		Extreme obesity	10/60 (17%)	360/2500 (14%)	0.58
During the trial	Jul 2012–Jun 2013	Obesity	59/88 (67%)	788/2853 (28%)	**<0.0001**
		Extreme obesity	52/75 (69%)	1376/2440 (56%)	**0.033**
	Jul 2013–Aug 2014	Obesity	85/86 (99%)	951/3113 (31%)	**<0.0001**
		Extreme obesity	51/64 (80%)	1585/2720 (58%)	**<0.001**
After the trial	Sep 2014–Sep 2015	Obesity	76/76 (100%)	1024/3220 (32%)	**<0.0001**
(ongoing programme)		Extreme obesity	44/55 (80%)	1637/2648 (62%)	**0.007**

*p*-values were derived from Fisher’s exact tests, with those statistically significant at *p* < 0.05 shown in bold.

## Data Availability

Due to the confidential and sensitive nature of the clinical audit data, public access to this dataset is restricted in accordance with the protection of participant health information. Explicit consent for the open sharing of this data was not obtained during the audit. Similarly, the dataset from the Whānau Pakari randomised clinical trial is not publicly available, owing to the stringent conditions set forth by the ethics approval. Nevertheless, access to both datasets may be granted upon a reasonable request, contingent upon receiving full ethics approval for the proposed study protocol and statistical analysis plan.

## Supplementary Materials

The following supporting information can be downloaded at https://www.mdpi.com/article/10.3390/children11020247/s1, Table S1: Referral rates to the Whānau Pakari programme among survey respondents who did or did not attend one of the Whānau Pakari referrer training half-day sessions. Table S2: Examples of feedback from health professionals who referred children and adolescents with overweight/obesity to the Whānau Pakari programme. Table S3: Recommendations from stakeholder feedback for ongoing programme development and potential transferability of the Whānau Pakari programme.
