# Supplementary material for: A Multisource Process Evaluation of a Community-Based Healthy Lifestyle Programme for Child and Adolescent Obesity
Source: Children (Basel). 2024 Feb 15;11(2):247. doi: 10.3390/children11020247 (PMC10887184; doi:10.3390/children11020247)
Supplement: Supplementary file 1 [file children-11-00247-s001.zip › children-2798656-supplementary.pdf]

Supplementary Table S1

Referral rates to the Whānau Pakari programme among survey respondents who did or did not attend one of the Whānau Pakari referrer training half-day sessions.

Referral to Whānau Pakari	Did not attend	Attended	*p*-value
n	85	21	
**Referred**	34 (40%)	2 (10%)	0.009
**Did not refer**	51 (60%)	19 (90%)	

Data are n (%). The *p*-value was derived from a Fisher's exact test.

Supplementary Table S2

Examples of feedback from health professionals who referred children and adolescents with overweight/obesity to the Whānau Pakari programme.

Positive experiences

"It is definitely easier now to discuss a child's weight issues with a parent since we have a positive support programme to offer them."

"Whānau Pakari made help more accessible for our practice, was already trying to help families with varying degrees of success. Much better having continuity."

"…I feel the most significant thing Whānau Pakari has contributed to is an increased awareness of weight/healthy eating and its involvement in the prevention of future chronic illness."

Challenges

"Clearly the aims and intentions of Whānau Pakari are positive. In moving forward, I think we need to work more on the motivational aspects of childhood obesity. I feel we are still coming across too many families who agree to referral and then do not continue with the programme. This is for a variety of reasons, I'm sure, but if we can identify those and act on them, Whānau Pakari will become more successful."

"Great service from our point of view. Still the patients that need most help the hardest to get to engage."

"Whānau Pakari suits a certain type of person. Not all families ready to take on the challenge but the ones that do benefit from the course."

"Unfortunately, the clients I have referred over the last 3 years start off really well but due to various reasons discontinue after 3-6 months.

Supplementary Table S3

Recommendations from stakeholder feedback for ongoing programme development and potential transferability of the Whānau Pakari programme.

- The healthy lifestyle coordinator, physical activity coordinator, dietitian, psychologist, and paediatrician are all required roles within the multi-disciplinary team.
- The stakeholders and Whānau Pakari team are recommended to continue to work closely with Māori health providers to involve Māori expertise and support strong community linkages. More Māori involvement as part of the service delivery team and at the governance level is recommended.
- Overall, the multi-disciplinary team worked well together, and it is recommended that the service design for this aspect of the programme be continued, with its emphasis on strong communication, a cohesive team structure, fortnightly meetings, and clearly defined responsibilities among the different team members.
- Whānau Pakari needs to be seen as a blended programme of non-medical and health/wellbeing support to achieve the programme's strategic objectives of reaching and engaging with Māori families and providing clinical services to all participants effectively.
- Home visits and the 'demedicalisation' of the delivered services are most likely critical to the success of Whānau Pakari and should continue to be a feature of the programme.
- Offering different sessions by age group should be considered by stakeholders and the Whānau Pakari team to tailor content better.
- The referral process worked well and should continue in its current form.
